# [6,6′-Bis(1,1-di­methyl­eth­yl)-4,4′-dimethyl-2,2′-methyl­enediphenolato-κ^2^ *O*,*O*′]di­chlorido­(9*H*-fluoren-9-ol-κ*O*)titanium(IV)–fluorene–diethyl ether (1/0.5/1)

**DOI:** 10.1107/S1600536813030249

**Published:** 2013-11-20

**Authors:** Alastair J. Nielson, Chaohong Shen, Joyce M. Waters

**Affiliations:** a Chemistry, Institute of Natural and Mathematical Sciences, Massey University at Albany, PO Box 102904 North Shore Mail Centre, Auckland, New Zealand; b Institute of Fundamental Sciences - Chemistry, Massey University at Albany, PO Box 102904 North Shore Mail Centre, Auckland, New Zealand

## Abstract

The title adduct, [TiCl_2_(C_23_H_30_O_2_)(C_13_H_10_O]·0.5C_13_H_10_·C_4_H_10_O, is a monomer with a trigonal–bypyramidal coordination sphere of the Ti^IV^ atom in which the ligand O atoms of the bidentate diphenolate anion are located in both apical and equatorial positions. Chloride ligands occupy the remaining two equatorial sites of the trigonal bypyramid with the fluoren-9-ol O atom occupying the other apical site. The hy­droxy group H atom of this latter group is hydrogen bonded to an O atom of a non-coordinating diethyl ether mol­ecule. The title compound also contains a further fluorene solvent mol­ecule, which lies across a centre of symmetry and which is equally disordered over an inversion centre.

## Related literature

For monomeric complexes of 4-coordinate titanium contain­ing the 2,2′-methyl­ene-bis-(4-methyl-6-*tert*-butyl­phen­o­lato) ligand, see: Toscano *et al.* (1998 ▶). For two other structures with a five-coordinate metal atom containing this type of ligand, see: Okuda *et al.* (1995 ▶); Gielens *et al.* (1999 ▶).
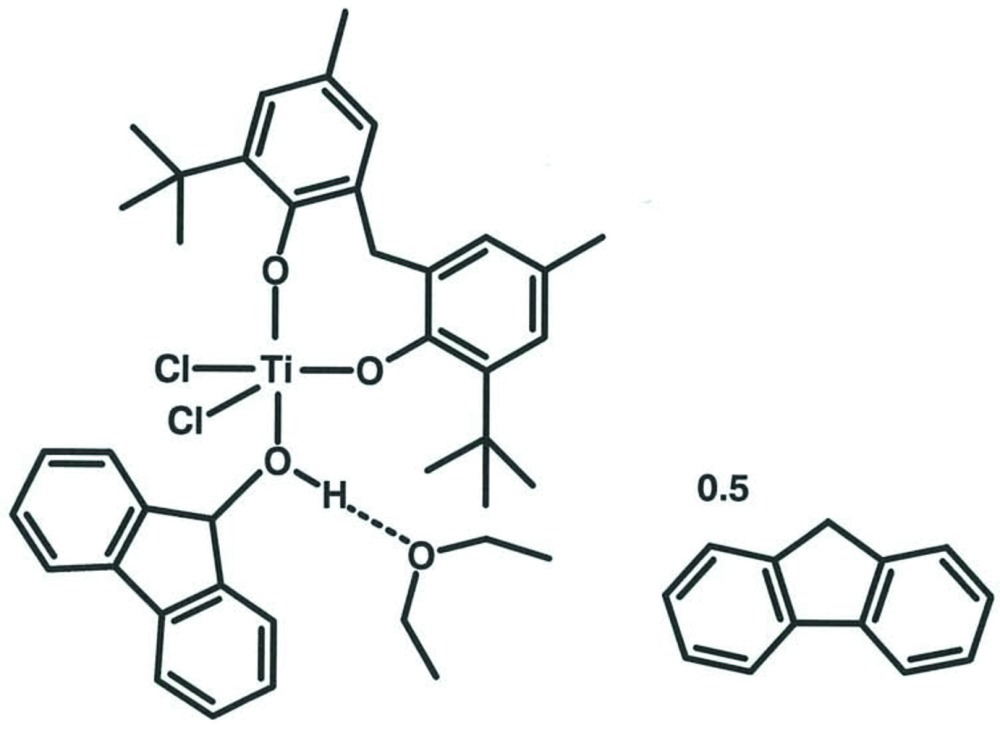


## Experimental

### 

#### Crystal data

[TiCl_2_(C_23_H_30_O_2_)(C_13_H_10_O)]·C_4_H_10_O·0.5C_13_H_10_
*M* *_r_* = 796.71
Triclinic, 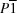

*a* = 12.710 (3) Å
*b* = 13.227 (3) Å
*c* = 14.431 (3) Å
α = 110.28 (3)°
β = 109.22 (3)°
γ = 91.72 (3)°
*V* = 2120.2 (7) Å^3^
*Z* = 2
Mo *K*α radiation
μ = 0.37 mm^−1^
*T* = 203 K
0.28 × 0.22 × 0.08 mm

#### Data collection

Siemens SMART diffractometer
Absorption correction: multi-scan (Blessing, 1995 ▶) *T*_min_ = 0.904, *T*_max_ = 0.971
18146 measured reflections
7432 independent reflections
6033 reflections with *I* > 2σ(*I*)
*R*_int_ = 0.025

#### Refinement

*R*[*F* ^2^ > 2σ(*F* ^2^)] = 0.036
*wR*(*F* ^2^) = 0.086
*S* = 1.07
7432 reflections
531 parameters
H atoms treated by a mixture of independent and constrained refinement
Δρ_max_ = 0.35 e Å^−3^
Δρ_min_ = −0.37 e Å^−3^

### 

Data collection: *SMART* (Siemens, 1995 ▶); cell refinement: *SAINT* (Siemens, 1995 ▶); data reduction: *SAINT*; program(s) used to solve structure: *SHELXS90* (Sheldrick, 2008 ▶); program(s) used to refine structure: *SHELXL97* (Sheldrick, 2008 ▶); molecular graphics: *ORTEP-3 for Windows* (Farrugia, 1999 ▶); software used to prepare material for publication: *SHELXTL* (Sheldrick, 2008 ▶).

## Supplementary Material

Crystal structure: contains datablock(s) I, global. DOI: 10.1107/S1600536813030249/bv2226sup1.cif

Structure factors: contains datablock(s) I. DOI: 10.1107/S1600536813030249/bv2226Isup2.hkl

Additional supplementary materials:  crystallographic information; 3D view; checkCIF report

## Figures and Tables

**Table 1:** Selected bond lengths (Å)

Ti—O2	1.7755 (13)
Ti—O1	1.8040 (14)
Ti—O4	2.1485 (15)
Ti—Cl1	2.2778 (12)
Ti—Cl2	2.2963 (9)

**Table 2:** Hydrogen-bond geometry (Å, °)

*D*—H⋯*A*	*D*—H	H⋯*A*	*D*⋯*A*	*D*—H⋯*A*
O4—H4⋯O3	0.89 (3)	1.76 (3)	2.648 (2)	180 (3)

**Fig. 1.**
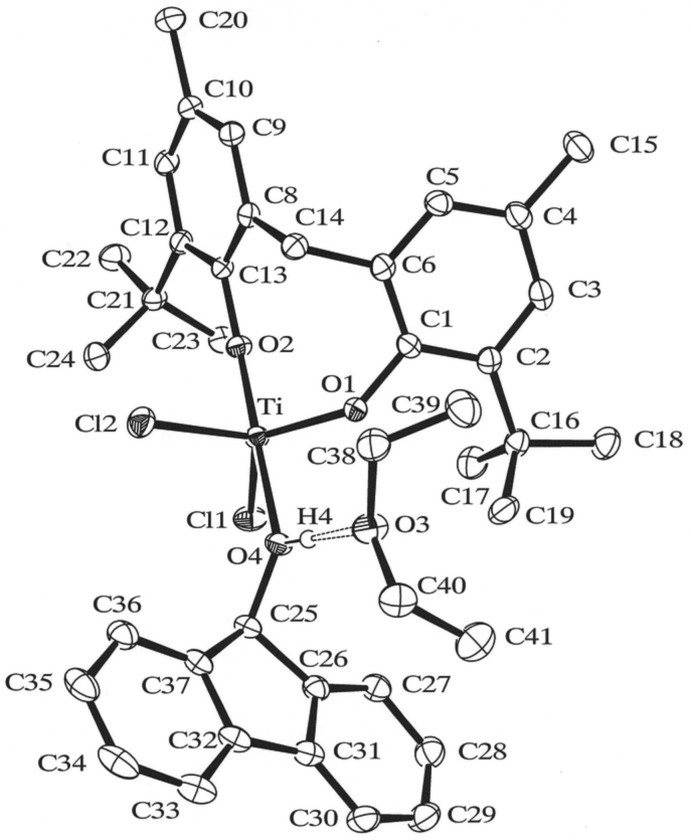
Diagram of title compound showing atom labeling and with atomic displacement parameters drawnn at the 50% probability level. Hydrogen atoms are omitted for clarity.

## supplementary crystallographic information

### 1. Comment

Titanium complexes containing the 2,2-methylene*bis*-(6-*tert*-butyl-4-methylphenolato) ligand (OAr)_2_ are well known and have been used as catalysts for olefin polymerization reactions. These catalysts are normally based on the dichloro complex [TiCl_2_{(OAr)_2_}] for which the X-ray crystal structures of this type of molecule shows a monomeric molecule with a distorted tetrahedral geometry (Toscano *et al.* 1998). This type of molecule is apparently coordinatively unsaturated since there are two reports of X-ray structures which show a fifth ligand can be added (Okuda *et al.* 1995; Gielens *et al.* 1999). During reactions of [TiCl_2_{(OAr)_2_}] in which we were attempting to replace one of the chloro ligands with a fluorene ligand C_13_H_9_, (Fl), the lithiated salt of fluorene was reacted with the titanium precursor and the resulting solution was stood for several months at -20° in an attempt to form crystals of the expected [TiCl(Fl){(OAr)_2_}] product. Over the extended crystallization period a small quantity of crystals was obtained and these were found to be the fluoren-9-ol adduct of the original [TiCl_2_{(OAr)_2_}] starting material with solvate molecules of diethyl ether and fluorene included. The fluoren-9-ol ligand apparently resulted from the lithiated fluorene undergoing hydrolysis as a result of slow moisture ingress into the reaction flask over the crystallization period and the resulting alcohol molecule coordinating to unreacted [TiCl_2_{(OAr)_2_}]. The small amount of crystalline material obtained supports this hypothesis. The fluorene molecule found in the unit cell apparently arises from the initial reaction of fluorene with *n*-butyl lithium not reaching completion.

The overall coordination geometry of the molecule is that of a distorted trigonal bipyramid with the oxygen atom of one side of the bidentate *bis*-phenolato ligand in one apical position and the oxygen atom of the fluoren-9-ol ligand in the other apical site. This positioning is similar to that of the two other 5-coordinate molecules characterized by X-ray crystallography (Okuda *et al.* 1995; Gielens *et al.*1999). The second oxygen atom of the bidentate *bis*-phenolato ligand occupies one of the equatorial sites with the remaining equatorial positions being taken up by chloro ligands. The largest distortion of the trigonal bipyramid essentially arises from the positioning of the *tert*-butyl group in the 2-position of the benzene ring of the phenolato ligand that lies in the apical position. This group lies over the top of Cl(1) which apparently causes the O(2)—Ti—Cl(1) bond angle to open out to 98.5 (1)°. In comparison Cl(2) is not in close proximity to the *tert*-butyl group and the O(2)—Ti—Cl(2) bond angle is smaller at 91.9 (1)°. The O(2)—Ti—O(1) bond angle which involves the bidentate *bis*-phenolato ligand is 95.6 (1)°. On the opposite side of the molecule where the fluorene-9-ol ligand resides the O(4)—Ti-ligand angles are all somewhat compressed with values ranging from 82.3 (1) to 87.1 (1)°. The angles associated with the equatorial sites of the trigonal bipyramid [range 117.46 (4) to 121.8 (1) °] show little, if any, compression effects. In this case the *tert*-butyl group in the 2-position of the phenolato ligand attached to the equatorial site, O(1) is well away from Cl(1), Cl(2) and O(4).

For the Ti—O bond distances associated with the bidentate *bis*-phenolato ligand, the Ti—O(2) bond length [1.776 (1) Å] which involves the oxygen in the apical position of the trigonal bipyramid is significantly shorter than the Ti—O(1) bond length [1.804 (1) Å] which involves the equatorial position. The shorter bond to O(2) is related to a greater degree of π-donation from oxygen lone pairs to the metal. Features associated with this aspect are that O(2) lies *trans* to the datively bonded O(4) atom which means there is minimal competition for π-orbitals on the metal. In addition the Ti—O(2)—C(13) bond angle [155.3 (1)°] approaches linearity which allows a maximum donation of the oxygen atom p orbitals. For the longer Ti—O(1) bond, the Ti—O(1)—C(1) bond angle is 140.5 (1)° which allows less donation of this type from the oxygen. The bidentate ligand is positioned with the carbon atom that forms the bridge between the two phenyl rings (C14) pointing towards the back of the molecule and it is this arrangement that places the *tert*-butyl group at the 2-postion of the apical phenolato ligand phenyl group over Cl(1).

The fluoren-9-ol ligand coordinates to the metal centre *via* a dative bond involving a lone pair of electrons from the alcohol oxygen O(4). Since the bond does not have a π-donor component the Ti—O(4) bond length [2.149 (2) Å] is much longer than the *bis*-phenolato ligand Ti—O bond lengths. The Ti—O(4)—C(25) bond angle is 130.8 (1)° which is larger than the normal bent angle and reflects the push-back effect oxygen lone pairs have on this type of molecule. With this angle there is no clash between the C(25) hydrogen and the nearby Cl(1) atom.

The alcohol hydrogen (H4) is hydrogen bonded to the oxygen atom of a non-coordinated diethyl ether molecule at a distance of 1.76 (3) Å in a linear arrangement [O(4)—H(4)···O(3) bond angle 180 (3)°]. The diethyl ether C(38)—O(3)—C(40) bond angle is 115.3 (2)°.

The crystal structure also shows that there is also a half-weighted fluorene molecule, C_13_H_10_ contained in the unit cell which is not coordinated to the metal. This lies across a centre of symmetry.

### 2. Experimental

Using normal bench-top techniques for air-sensitive compounds, *n*-butyl lithium (3.8 ml of a 1.6 mol/*L* solution, 6.02 mmol) was added dropwise to a solution of fluorene (1.00 g, 6.02 mmol) in diethyl ether (30 ml) cooled to 78°C and the mixture was warmed to room temperature and stirred for a further 2 h. The solution was added dropwise to a solution of [TiCl_2_{(OAr)_2_}] (2.83 g, 6.02 mmol) in diethyl ether (50 ml), chilled to 0°C and the mixture allowed to warm to room temperature and the stirring continued overnight. The solution was filtered, the volume reduced to *ca* 20 ml and the solution stood at -20° C for several months whereupon a small quantity of colourless crystals was formed. A crystal was chosen from the mass and the X-ray crystal structure obtained.

### 3. Refinement

All H atoms, except H4, were included in calculated positions and refined using a riding model [*U*(H)_eq_ = 1.2*U*C_eq_ for aromatic CH and *U*(H) = 1.5*U*(C) for methyl H atoms]. C—H distances of 0.96 Å and 0.93 Å were assumed for aromatic and methyl groups respectively. For H4, the atom involved in the H-bonding, the positional parameters were refined but the thermal parameter was held constant at 0.08.

The non H-bonded solvent molecule was half-weighted since it lay across the centre of symmetry and its two six-membered rings were refined as rigid groups.

### Crystal data

**Table:**

[TiCl_2_(C_23_H_30_O_2_)(C_13_H_10_O)]·C_4_H_10_O·0.5C_13_H_10_	*Z* = 2
*M**_r_* = 796.71	*F*(000) = 844
Triclinic, *P*1	*D*_x_ = 1.248 Mg m^−^^3^
Hall symbol: -P 1	Mo *K*α radiation, λ = 0.71073 Å
*a* = 12.710 (3) Å	Cell parameters from 5551 reflections
*b* = 13.227 (3) Å	θ = 2–25°
*c* = 14.431 (3) Å	µ = 0.37 mm^−^^1^
α = 110.28 (3)°	*T* = 203 K
β = 109.22 (3)°	Irregular fragment, red
γ = 91.72 (3)°	0.28 × 0.22 × 0.08 mm
*V* = 2120.2 (7) Å^3^	

### Data collection

**Table:**

Siemens SMART diffractometer	7432 independent reflections
Radiation source: fine-focus sealed tube	6033 reflections with *I* > 2σ(*I*)
Graphite monochromator	*R*_int_ = 0.025
Area detector ω scans	θ_max_ = 25.1°, θ_min_ = 1.6°
Absorption correction: multi-scan (Blessing, 1995)	*h* = −15→14
*T*_min_ = 0.904, *T*_max_ = 0.971	*k* = −15→14
18146 measured reflections	*l* = 0→17

### Refinement

**Table:**

Refinement on *F*^2^	Secondary atom site location: difference Fourier map
Least-squares matrix: full	Hydrogen site location: inferred from neighbouring sites
*R*[*F*^2^ > 2σ(*F*^2^)] = 0.036	H atoms treated by a mixture of independent and constrained refinement
*wR*(*F*^2^) = 0.086	*w* = 1/[σ^2^(*F*_o_^2^) + (0.0268*P*)^2^ + 1.2664*P*] where *P* = (*F*_o_^2^ + 2*F*_c_^2^)/3
*S* = 1.07	(Δ/σ)_max_ = 0.001
7432 reflections	Δρ_max_ = 0.35 e Å^−^^3^
531 parameters	Δρ_min_ = −0.37 e Å^−^^3^
0 restraints	Extinction correction: *SHELXL97* (Sheldrick, 2008), Fc^*^=kFc[1+0.001xFc^2^λ^3^/sin(2θ)]^-1/4^
0 constraints	Extinction coefficient: 0.0008 (3)
Primary atom site location: structure-invariant direct methods	

### Special details

**Table:**

Geometry. All e.s.d.'s (except the e.s.d. in the dihedral angle between two l.s. planes) are estimated using the full covariance matrix. The cell e.s.d.'s are taken into account individually in the estimation of e.s.d.'s in distances, angles and torsion angles; correlations between e.s.d.'s in cell parameters are only used when they are defined by crystal symmetry. An approximate (isotropic) treatment of cell e.s.d.'s is used for estimating e.s.d.'s involving l.s. planes.
Refinement. Refinement of *F*^2^ against ALL reflections. The weighted *R*-factor *wR* and goodness of fit *S* are based on *F*^2^, conventional *R*-factors *R* are based on *F*, with *F* set to zero for negative *F*^2^. The threshold expression of *F*^2^ > σ(*F*^2^) is used only for calculating *R*-factors(gt) *etc*. and is not relevant to the choice of reflections for refinement. *R*-factors based on *F*^2^ are statistically about twice as large as those based on *F*, and *R*- factors based on ALL data will be even larger.

### Fractional atomic coordinates and isotropic or equivalent isotropic displacement parameters (Å^2^)

**Table:**

	*x*	*y*	*z*	*U*_iso_*/*U*_eq_	Occ. (<1)
Ti	0.59181 (3)	0.73459 (3)	0.24921 (3)	0.01990 (10)	
Cl1	0.66189 (4)	0.84629 (4)	0.42383 (4)	0.03050 (13)	
Cl2	0.64541 (5)	0.56538 (4)	0.20349 (4)	0.03257 (14)	
O4	0.46553 (12)	0.65866 (11)	0.28390 (11)	0.0232 (3)	
O1	0.46954 (11)	0.76509 (11)	0.16236 (10)	0.0218 (3)	
O2	0.69091 (11)	0.78731 (11)	0.20806 (10)	0.0215 (3)	
O3	0.26771 (12)	0.56947 (13)	0.12867 (11)	0.0341 (4)	
C1	0.44084 (16)	0.81060 (16)	0.08647 (15)	0.0210 (4)	
C2	0.37136 (16)	0.89285 (16)	0.09469 (16)	0.0230 (4)	
C3	0.34059 (17)	0.93113 (17)	0.01229 (17)	0.0275 (5)	
H3	0.2942	0.9853	0.0154	0.033*	
C4	0.37470 (17)	0.89349 (18)	−0.07426 (16)	0.0278 (5)	
C5	0.44411 (17)	0.81449 (17)	−0.07777 (16)	0.0267 (5)	
H5	0.4687	0.7888	−0.1352	0.032*	
C6	0.47851 (16)	0.77200 (16)	0.00151 (15)	0.0221 (4)	
C8	0.68002 (17)	0.73915 (16)	0.03058 (15)	0.0219 (4)	
C9	0.73235 (17)	0.73767 (17)	−0.04085 (16)	0.0250 (4)	
H9	0.6906	0.7056	−0.1140	0.030*	
C10	0.84534 (18)	0.78286 (17)	−0.00581 (16)	0.0260 (5)	
C11	0.90583 (17)	0.83031 (17)	0.10316 (16)	0.0265 (5)	
H11	0.9821	0.8610	0.1267	0.032*	
C12	0.85864 (16)	0.83449 (16)	0.17901 (15)	0.0220 (4)	
C13	0.74406 (16)	0.78770 (16)	0.13949 (15)	0.0205 (4)	
C14	0.55725 (16)	0.68790 (16)	−0.00806 (15)	0.0235 (4)	
H14A	0.5503	0.6444	0.0331	0.028*	
H14B	0.5339	0.6383	−0.0824	0.028*	
C15	0.3383 (2)	0.9391 (2)	−0.16034 (18)	0.0376 (6)	
H15A	0.3996	0.9922	−0.1501	0.056*	
H15B	0.2729	0.9741	−0.1573	0.056*	
H15C	0.3191	0.8802	−0.2289	0.056*	
C16	0.33479 (18)	0.94173 (17)	0.19089 (17)	0.0271 (5)	
C17	0.4393 (2)	1.00425 (19)	0.29056 (18)	0.0367 (5)	
H17A	0.4159	1.0373	0.3504	0.055*	
H17B	0.4773	1.0608	0.2790	0.055*	
H17C	0.4904	0.9540	0.3051	0.055*	
C18	0.2532 (2)	1.02337 (19)	0.17562 (19)	0.0363 (5)	
H18A	0.1874	0.9872	0.1117	0.054*	
H18B	0.2910	1.0842	0.1695	0.054*	
H18C	0.2296	1.0502	0.2361	0.054*	
C19	0.2731 (2)	0.85313 (19)	0.2091 (2)	0.0356 (5)	
H19A	0.2559	0.8859	0.2727	0.053*	
H19B	0.3208	0.7986	0.2172	0.053*	
H19C	0.2034	0.8188	0.1488	0.053*	
C20	0.90321 (19)	0.7785 (2)	−0.08351 (18)	0.0347 (5)	
H20A	0.9370	0.8518	−0.0689	0.052*	
H20B	0.8481	0.7479	−0.1552	0.052*	
H20C	0.9615	0.7331	−0.0761	0.052*	
C21	0.92739 (16)	0.88579 (17)	0.29859 (15)	0.0239 (4)	
C22	1.04921 (18)	0.9323 (2)	0.32137 (18)	0.0347 (5)	
H22A	1.0869	0.8736	0.2918	0.052*	
H22B	1.0891	0.9668	0.3974	0.052*	
H22C	1.0488	0.9858	0.2893	0.052*	
C23	0.87372 (18)	0.98044 (17)	0.35278 (16)	0.0277 (5)	
H23A	0.8765	1.0376	0.3255	0.042*	
H23B	0.9151	1.0095	0.4287	0.042*	
H23C	0.7957	0.9539	0.3383	0.042*	
C24	0.93313 (18)	0.79741 (18)	0.34662 (17)	0.0297 (5)	
H24A	0.8573	0.7678	0.3357	0.045*	
H24B	0.9772	0.8296	0.4220	0.045*	
H24C	0.9685	0.7393	0.3123	0.045*	
C25	0.47153 (17)	0.63855 (16)	0.37816 (15)	0.0237 (4)	
H25	0.5481	0.6691	0.4325	0.080*	
C26	0.38535 (17)	0.69044 (17)	0.42380 (15)	0.0242 (4)	
C27	0.37082 (18)	0.79872 (18)	0.45693 (16)	0.0293 (5)	
H27	0.4159	0.8516	0.4504	0.035*	
C28	0.2877 (2)	0.8277 (2)	0.50039 (17)	0.0362 (5)	
H28	0.2763	0.9008	0.5233	0.043*	
C29	0.2217 (2)	0.7490 (2)	0.50998 (18)	0.0391 (6)	
H29	0.1664	0.7696	0.5398	0.047*	
C30	0.23619 (19)	0.6406 (2)	0.47630 (18)	0.0357 (5)	
H30	0.1908	0.5878	0.4827	0.043*	
C31	0.31854 (18)	0.61101 (17)	0.43305 (16)	0.0275 (5)	
C32	0.35526 (18)	0.50511 (17)	0.39458 (16)	0.0279 (5)	
C33	0.3197 (2)	0.40365 (18)	0.39237 (17)	0.0355 (5)	
H33	0.2589	0.3934	0.4134	0.043*	
C34	0.3756 (2)	0.31783 (18)	0.35860 (17)	0.0390 (6)	
H34	0.3522	0.2489	0.3569	0.047*	
C35	0.4651 (2)	0.33196 (18)	0.32742 (17)	0.0374 (6)	
H35	0.5026	0.2730	0.3060	0.045*	
C36	0.5004 (2)	0.43296 (18)	0.32745 (16)	0.0313 (5)	
H36	0.5605	0.4425	0.3054	0.038*	
C37	0.44433 (17)	0.51884 (16)	0.36079 (15)	0.0248 (4)	
C38	0.2640 (2)	0.53601 (19)	0.02120 (17)	0.0347 (5)	
H38A	0.2390	0.4569	−0.0145	0.042*	
H38B	0.3404	0.5517	0.0224	0.042*	
C39	0.1865 (2)	0.5920 (2)	−0.04151 (18)	0.0425 (6)	
H39A	0.1107	0.5770	−0.0431	0.064*	
H39B	0.1860	0.5651	−0.1133	0.064*	
H39C	0.2129	0.6702	−0.0086	0.064*	
C40	0.1719 (2)	0.52214 (19)	0.14099 (19)	0.0368 (5)	
H40A	0.1980	0.4828	0.1886	0.044*	
H40B	0.1214	0.4695	0.0720	0.044*	
C41	0.1082 (2)	0.6081 (2)	0.1853 (2)	0.0448 (6)	
H41A	0.1600	0.6648	0.2494	0.067*	
H41B	0.0506	0.5754	0.2015	0.067*	
H41C	0.0728	0.6397	0.1335	0.067*	
C51	1.0508 (6)	0.5759 (4)	0.6109 (5)	0.049 (2)	0.50
C52	1.0701 (6)	0.6721 (5)	0.6982 (4)	0.068 (3)	0.50
H52	1.1242	0.6805	0.7643	0.082*	0.50
C53	1.0092 (8)	0.7557 (5)	0.6880 (5)	0.059 (7)	0.50
H53	1.0223	0.8207	0.7470	0.071*	0.50
C54	0.9291 (6)	0.7431 (4)	0.5903 (5)	0.051 (2)	0.50
H54	0.8880	0.7997	0.5834	0.062*	0.50
C55	0.9098 (5)	0.6470 (5)	0.5030 (4)	0.0468 (16)	0.50
H55	0.8556	0.6385	0.4370	0.056*	0.50
C56	0.9706 (6)	0.5634 (4)	0.5133 (4)	0.044 (2)	0.50
C57	0.9651 (6)	0.4566 (3)	0.4346 (5)	0.0395 (19)	0.50
C58	0.8984 (5)	0.4057 (5)	0.3278 (5)	0.0487 (15)	0.50
H58	0.8449	0.4420	0.2947	0.058*	0.50
C59	0.9108 (7)	0.3012 (5)	0.2699 (4)	0.058 (2)	0.50
H59	0.8657	0.2668	0.1977	0.070*	0.50
C60	0.9899 (8)	0.2476 (5)	0.3188 (6)	0.072 (9)	0.50
H60	0.9982	0.1770	0.2797	0.086*	0.50
C61	1.0565 (6)	0.2986 (5)	0.4256 (6)	0.065 (3)	0.50
H61	1.1100	0.2623	0.4586	0.078*	0.50
C62	1.0441 (5)	0.4030 (5)	0.4835 (4)	0.0428 (19)	0.50
C63	1.1025 (5)	0.4738 (5)	0.5994 (5)	0.0620 (17)	0.50
H63A	1.0880	0.4385	0.6445	0.074*	0.50
H63B	1.1843	0.4897	0.6177	0.074*	0.50
H4	0.399 (3)	0.629 (3)	0.232 (3)	0.080*	

### Atomic displacement parameters (Å^2^)

**Table:**

	*U*^11^	*U*^22^	*U*^33^	*U*^12^	*U*^13^	*U*^23^
Ti	0.01863 (19)	0.0224 (2)	0.01971 (19)	0.00279 (14)	0.00761 (15)	0.00857 (15)
Cl1	0.0317 (3)	0.0332 (3)	0.0217 (3)	−0.0040 (2)	0.0094 (2)	0.0055 (2)
Cl2	0.0367 (3)	0.0276 (3)	0.0404 (3)	0.0119 (2)	0.0207 (3)	0.0142 (2)
O4	0.0222 (7)	0.0301 (8)	0.0208 (7)	0.0024 (6)	0.0077 (6)	0.0137 (6)
O1	0.0198 (7)	0.0254 (7)	0.0237 (7)	0.0056 (6)	0.0090 (6)	0.0123 (6)
O2	0.0202 (7)	0.0250 (7)	0.0196 (7)	0.0020 (6)	0.0082 (6)	0.0079 (6)
O3	0.0273 (8)	0.0429 (9)	0.0268 (8)	−0.0019 (7)	0.0076 (7)	0.0096 (7)
C1	0.0167 (10)	0.0234 (11)	0.0213 (10)	−0.0009 (8)	0.0032 (8)	0.0105 (9)
C2	0.0189 (10)	0.0220 (10)	0.0278 (11)	0.0016 (8)	0.0075 (9)	0.0102 (9)
C3	0.0228 (11)	0.0257 (11)	0.0347 (12)	0.0050 (9)	0.0066 (9)	0.0159 (10)
C4	0.0228 (11)	0.0332 (12)	0.0285 (11)	0.0016 (9)	0.0053 (9)	0.0168 (10)
C5	0.0241 (11)	0.0334 (12)	0.0215 (10)	0.0023 (9)	0.0068 (9)	0.0104 (9)
C6	0.0172 (10)	0.0233 (11)	0.0225 (10)	0.0005 (8)	0.0041 (8)	0.0079 (9)
C8	0.0229 (11)	0.0218 (10)	0.0229 (10)	0.0074 (8)	0.0094 (9)	0.0095 (9)
C9	0.0274 (11)	0.0278 (11)	0.0203 (10)	0.0086 (9)	0.0096 (9)	0.0083 (9)
C10	0.0303 (12)	0.0292 (11)	0.0261 (11)	0.0116 (9)	0.0158 (9)	0.0135 (9)
C11	0.0208 (11)	0.0310 (12)	0.0318 (12)	0.0060 (9)	0.0120 (9)	0.0143 (10)
C12	0.0206 (10)	0.0226 (10)	0.0245 (10)	0.0054 (8)	0.0098 (8)	0.0094 (9)
C13	0.0227 (10)	0.0216 (10)	0.0224 (10)	0.0080 (8)	0.0119 (8)	0.0104 (9)
C14	0.0219 (11)	0.0258 (11)	0.0188 (10)	0.0025 (9)	0.0056 (8)	0.0056 (9)
C15	0.0375 (14)	0.0453 (14)	0.0352 (13)	0.0098 (11)	0.0094 (11)	0.0246 (12)
C16	0.0263 (11)	0.0241 (11)	0.0321 (12)	0.0072 (9)	0.0123 (9)	0.0102 (9)
C17	0.0372 (13)	0.0336 (13)	0.0334 (13)	0.0069 (11)	0.0117 (11)	0.0067 (11)
C18	0.0384 (14)	0.0314 (13)	0.0435 (14)	0.0137 (11)	0.0202 (11)	0.0134 (11)
C19	0.0369 (13)	0.0354 (13)	0.0495 (14)	0.0129 (11)	0.0292 (12)	0.0203 (12)
C20	0.0336 (13)	0.0469 (14)	0.0334 (12)	0.0111 (11)	0.0195 (11)	0.0193 (11)
C21	0.0180 (10)	0.0303 (11)	0.0218 (10)	0.0023 (9)	0.0067 (8)	0.0087 (9)
C22	0.0225 (11)	0.0491 (15)	0.0306 (12)	−0.0006 (10)	0.0085 (10)	0.0142 (11)
C23	0.0264 (11)	0.0273 (11)	0.0246 (11)	0.0004 (9)	0.0068 (9)	0.0067 (9)
C24	0.0251 (11)	0.0347 (12)	0.0279 (11)	0.0064 (10)	0.0064 (9)	0.0130 (10)
C25	0.0280 (11)	0.0259 (11)	0.0203 (10)	0.0041 (9)	0.0093 (9)	0.0119 (9)
C26	0.0248 (11)	0.0285 (11)	0.0181 (10)	0.0035 (9)	0.0056 (8)	0.0093 (9)
C27	0.0319 (12)	0.0303 (12)	0.0245 (11)	0.0035 (10)	0.0090 (9)	0.0102 (10)
C28	0.0406 (14)	0.0370 (13)	0.0312 (12)	0.0139 (11)	0.0149 (11)	0.0107 (11)
C29	0.0345 (13)	0.0548 (16)	0.0339 (13)	0.0140 (12)	0.0191 (11)	0.0167 (12)
C30	0.0307 (13)	0.0473 (15)	0.0375 (13)	0.0046 (11)	0.0176 (11)	0.0210 (12)
C31	0.0280 (12)	0.0315 (12)	0.0237 (11)	0.0000 (9)	0.0084 (9)	0.0126 (9)
C32	0.0324 (12)	0.0294 (12)	0.0202 (10)	−0.0014 (9)	0.0058 (9)	0.0114 (9)
C33	0.0427 (14)	0.0345 (13)	0.0270 (11)	−0.0063 (11)	0.0091 (10)	0.0134 (10)
C34	0.0635 (17)	0.0238 (12)	0.0237 (11)	−0.0035 (11)	0.0078 (11)	0.0104 (10)
C35	0.0616 (17)	0.0271 (12)	0.0228 (11)	0.0115 (11)	0.0131 (11)	0.0103 (10)
C36	0.0404 (13)	0.0311 (12)	0.0231 (11)	0.0084 (10)	0.0115 (10)	0.0107 (10)
C37	0.0292 (11)	0.0254 (11)	0.0185 (10)	0.0015 (9)	0.0046 (9)	0.0107 (9)
C38	0.0379 (13)	0.0340 (13)	0.0287 (12)	0.0079 (10)	0.0120 (10)	0.0075 (10)
C39	0.0464 (15)	0.0434 (15)	0.0320 (13)	0.0093 (12)	0.0081 (11)	0.0133 (11)
C40	0.0349 (13)	0.0348 (13)	0.0365 (13)	−0.0035 (10)	0.0115 (11)	0.0108 (11)
C41	0.0367 (14)	0.0425 (15)	0.0484 (15)	0.0002 (12)	0.0163 (12)	0.0088 (12)
C51	0.042 (4)	0.062 (5)	0.049 (6)	−0.004 (4)	0.008 (4)	0.039 (5)
C52	0.080 (6)	0.076 (7)	0.038 (4)	−0.023 (6)	0.003 (4)	0.029 (4)
C53	0.065 (12)	0.065 (14)	0.060 (10)	−0.009 (10)	0.027 (9)	0.034 (9)
C54	0.058 (5)	0.052 (5)	0.062 (5)	0.003 (4)	0.028 (4)	0.037 (4)
C55	0.045 (4)	0.053 (4)	0.054 (4)	−0.003 (3)	0.023 (3)	0.029 (3)
C56	0.033 (4)	0.059 (5)	0.057 (5)	0.002 (3)	0.020 (4)	0.037 (4)
C57	0.032 (4)	0.051 (4)	0.046 (5)	0.000 (3)	0.017 (4)	0.029 (4)
C58	0.050 (4)	0.057 (4)	0.052 (4)	0.010 (3)	0.022 (3)	0.032 (4)
C59	0.073 (6)	0.050 (4)	0.056 (4)	−0.001 (4)	0.029 (4)	0.022 (4)
C60	0.11 (2)	0.045 (12)	0.088 (14)	0.015 (12)	0.069 (15)	0.031 (10)
C61	0.058 (5)	0.067 (7)	0.121 (10)	0.031 (5)	0.057 (6)	0.070 (7)
C62	0.035 (4)	0.053 (4)	0.060 (5)	0.007 (4)	0.018 (3)	0.044 (4)
C63	0.047 (3)	0.079 (4)	0.076 (4)	0.006 (3)	0.013 (3)	0.058 (4)

### Geometric parameters (Å, º)

**Table:**

Ti—O2	1.7755 (13)	C24—H24B	0.9700
Ti—O1	1.8040 (14)	C24—H24C	0.9700
Ti—O4	2.1485 (15)	C25—C26	1.520 (3)
Ti—Cl1	2.2778 (12)	C25—C37	1.525 (3)
Ti—Cl2	2.2963 (9)	C25—H25	0.9900
O4—C25	1.453 (2)	C26—C27	1.383 (3)
O4—H4	0.89 (3)	C26—C31	1.400 (3)
O1—C1	1.379 (2)	C27—C28	1.397 (3)
O2—C13	1.371 (2)	C27—H27	0.9400
O3—C38	1.442 (3)	C28—C29	1.389 (3)
O3—C40	1.442 (3)	C28—H28	0.9400
C1—C6	1.400 (3)	C29—C30	1.384 (3)
C1—C2	1.419 (3)	C29—H29	0.9400
C2—C3	1.395 (3)	C30—C31	1.387 (3)
C2—C16	1.543 (3)	C30—H30	0.9400
C3—C4	1.391 (3)	C31—C32	1.469 (3)
C3—H3	0.9400	C32—C33	1.389 (3)
C4—C5	1.386 (3)	C32—C37	1.402 (3)
C4—C15	1.509 (3)	C33—C34	1.387 (3)
C5—C6	1.395 (3)	C33—H33	0.9400
C5—H5	0.9400	C34—C35	1.384 (3)
C6—C14	1.520 (3)	C34—H34	0.9400
C8—C9	1.394 (3)	C35—C36	1.396 (3)
C8—C13	1.404 (3)	C35—H35	0.9400
C8—C14	1.519 (3)	C36—C37	1.387 (3)
C9—C10	1.391 (3)	C36—H36	0.9400
C9—H9	0.9400	C38—C39	1.504 (3)
C10—C11	1.396 (3)	C38—H38A	0.9800
C10—C20	1.517 (3)	C38—H38B	0.9800
C11—C12	1.398 (3)	C39—H39A	0.9700
C11—H11	0.9400	C39—H39B	0.9700
C12—C13	1.408 (3)	C39—H39C	0.9700
C12—C21	1.537 (3)	C40—C41	1.499 (3)
C14—H14A	0.9800	C40—H40A	0.9800
C14—H14B	0.9800	C40—H40B	0.9800
C15—H15A	0.9700	C41—H41A	0.9700
C15—H15B	0.9700	C41—H41B	0.9700
C15—H15C	0.9700	C41—H41C	0.9700
C16—C19	1.533 (3)	C51—C52	1.3900
C16—C17	1.539 (3)	C51—C56	1.3900
C16—C18	1.540 (3)	C51—C63	1.502 (8)
C17—H17A	0.9700	C52—C53	1.3900
C17—H17B	0.9700	C52—H52	0.9400
C17—H17C	0.9700	C53—C54	1.3900
C18—H18A	0.9700	C53—H53	0.9400
C18—H18B	0.9700	C54—C55	1.3900
C18—H18C	0.9700	C54—H54	0.9400
C19—H19A	0.9700	C55—C56	1.3900
C19—H19B	0.9700	C55—H55	0.9400
C19—H19C	0.9700	C56—C57	1.455 (5)
C20—H20A	0.9700	C57—C58	1.3900
C20—H20B	0.9700	C57—C62	1.3900
C20—H20C	0.9700	C58—C59	1.3900
C21—C22	1.533 (3)	C58—H58	0.9400
C21—C23	1.539 (3)	C59—C60	1.3900
C21—C24	1.545 (3)	C59—H59	0.9400
C22—H22A	0.9700	C60—C61	1.3900
C22—H22B	0.9700	C60—H60	0.9400
C22—H22C	0.9700	C61—C62	1.3900
C23—H23A	0.9700	C61—H61	0.9400
C23—H23B	0.9700	C62—C63	1.511 (8)
C23—H23C	0.9700	C63—H63A	0.9800
C24—H24A	0.9700	C63—H63B	0.9800
			
O2—Ti—O1	95.60 (6)	C21—C23—H23C	109.5
O2—Ti—O4	174.46 (6)	H23A—C23—H23C	109.5
O1—Ti—O4	82.26 (6)	H23B—C23—H23C	109.5
O2—Ti—Cl1	98.45 (6)	C21—C24—H24A	109.5
O1—Ti—Cl1	118.21 (6)	C21—C24—H24B	109.5
O4—Ti—Cl1	87.05 (5)	H24A—C24—H24B	109.5
O2—Ti—Cl2	91.89 (5)	C21—C24—H24C	109.5
O1—Ti—Cl2	121.81 (6)	H24A—C24—H24C	109.5
O4—Ti—Cl2	84.95 (5)	H24B—C24—H24C	109.5
Cl1—Ti—Cl2	117.46 (4)	O4—C25—C26	112.09 (16)
C25—O4—Ti	130.83 (12)	O4—C25—C37	115.09 (16)
C25—O4—H4	111 (2)	C26—C25—C37	102.78 (16)
Ti—O4—H4	118 (2)	O4—C25—H25	108.9
C1—O1—Ti	140.46 (12)	C26—C25—H25	108.9
C13—O2—Ti	155.26 (13)	C37—C25—H25	108.9
C38—O3—C40	115.25 (17)	C27—C26—C31	121.2 (2)
O1—C1—C6	118.93 (17)	C27—C26—C25	128.98 (19)
O1—C1—C2	119.33 (17)	C31—C26—C25	109.77 (18)
C6—C1—C2	121.71 (17)	C26—C27—C28	118.5 (2)
C3—C2—C1	116.33 (18)	C26—C27—H27	120.8
C3—C2—C16	120.95 (18)	C28—C27—H27	120.8
C1—C2—C16	122.67 (17)	C29—C28—C27	120.3 (2)
C4—C3—C2	123.62 (19)	C29—C28—H28	119.8
C4—C3—H3	118.2	C27—C28—H28	119.8
C2—C3—H3	118.2	C30—C29—C28	121.0 (2)
C5—C4—C3	117.88 (19)	C30—C29—H29	119.5
C5—C4—C15	121.5 (2)	C28—C29—H29	119.5
C3—C4—C15	120.6 (2)	C29—C30—C31	119.1 (2)
C4—C5—C6	121.94 (19)	C29—C30—H30	120.5
C4—C5—H5	119.0	C31—C30—H30	120.5
C6—C5—H5	119.0	C30—C31—C26	119.9 (2)
C5—C6—C1	118.50 (18)	C30—C31—C32	131.2 (2)
C5—C6—C14	117.93 (18)	C26—C31—C32	108.92 (18)
C1—C6—C14	123.56 (17)	C33—C32—C37	120.1 (2)
C9—C8—C13	118.40 (18)	C33—C32—C31	130.8 (2)
C9—C8—C14	121.00 (18)	C37—C32—C31	108.98 (18)
C13—C8—C14	120.59 (17)	C34—C33—C32	118.8 (2)
C10—C9—C8	121.20 (19)	C34—C33—H33	120.6
C10—C9—H9	119.4	C32—C33—H33	120.6
C8—C9—H9	119.4	C35—C34—C33	121.1 (2)
C9—C10—C11	118.48 (19)	C35—C34—H34	119.4
C9—C10—C20	121.22 (19)	C33—C34—H34	119.4
C11—C10—C20	120.28 (19)	C34—C35—C36	120.7 (2)
C10—C11—C12	123.26 (19)	C34—C35—H35	119.7
C10—C11—H11	118.4	C36—C35—H35	119.7
C12—C11—H11	118.4	C37—C36—C35	118.3 (2)
C11—C12—C13	116.04 (18)	C37—C36—H36	120.8
C11—C12—C21	122.33 (18)	C35—C36—H36	120.8
C13—C12—C21	121.63 (17)	C36—C37—C32	121.00 (19)
O2—C13—C8	117.27 (17)	C36—C37—C25	129.19 (19)
O2—C13—C12	120.12 (17)	C32—C37—C25	109.54 (18)
C8—C13—C12	122.61 (18)	O3—C38—C39	113.16 (19)
C8—C14—C6	112.87 (16)	O3—C38—H38A	108.9
C8—C14—H14A	109.0	C39—C38—H38A	108.9
C6—C14—H14A	109.0	O3—C38—H38B	108.9
C8—C14—H14B	109.0	C39—C38—H38B	108.9
C6—C14—H14B	109.0	H38A—C38—H38B	107.8
H14A—C14—H14B	107.8	C38—C39—H39A	109.5
C4—C15—H15A	109.5	C38—C39—H39B	109.5
C4—C15—H15B	109.5	H39A—C39—H39B	109.5
H15A—C15—H15B	109.5	C38—C39—H39C	109.5
C4—C15—H15C	109.5	H39A—C39—H39C	109.5
H15A—C15—H15C	109.5	H39B—C39—H39C	109.5
H15B—C15—H15C	109.5	O3—C40—C41	111.31 (19)
C19—C16—C17	109.86 (18)	O3—C40—H40A	109.4
C19—C16—C18	106.62 (17)	C41—C40—H40A	109.4
C17—C16—C18	107.34 (18)	O3—C40—H40B	109.4
C19—C16—C2	111.66 (17)	C41—C40—H40B	109.4
C17—C16—C2	109.51 (17)	H40A—C40—H40B	108.0
C18—C16—C2	111.72 (17)	C40—C41—H41A	109.5
C16—C17—H17A	109.5	C40—C41—H41B	109.5
C16—C17—H17B	109.5	H41A—C41—H41B	109.5
H17A—C17—H17B	109.5	C40—C41—H41C	109.5
C16—C17—H17C	109.5	H41A—C41—H41C	109.5
H17A—C17—H17C	109.5	H41B—C41—H41C	109.5
H17B—C17—H17C	109.5	C52—C51—C56	120.0
C16—C18—H18A	109.5	C52—C51—C63	131.3 (5)
C16—C18—H18B	109.5	C56—C51—C63	108.7 (5)
H18A—C18—H18B	109.5	C51—C52—C53	120.0
C16—C18—H18C	109.5	C51—C52—H52	120.0
H18A—C18—H18C	109.5	C53—C52—H52	120.0
H18B—C18—H18C	109.5	C54—C53—C52	120.0
C16—C19—H19A	109.5	C54—C53—H53	120.0
C16—C19—H19B	109.5	C52—C53—H53	120.0
H19A—C19—H19B	109.5	C53—C54—C55	120.0
C16—C19—H19C	109.5	C53—C54—H54	120.0
H19A—C19—H19C	109.5	C55—C54—H54	120.0
H19B—C19—H19C	109.5	C54—C55—C56	120.0
C10—C20—H20A	109.5	C54—C55—H55	120.0
C10—C20—H20B	109.5	C56—C55—H55	120.0
H20A—C20—H20B	109.5	C55—C56—C51	120.0
C10—C20—H20C	109.5	C55—C56—C57	129.9 (7)
H20A—C20—H20C	109.5	C51—C56—C57	110.1 (7)
H20B—C20—H20C	109.5	C58—C57—C62	120.0
C22—C21—C12	111.68 (17)	C58—C57—C56	131.9 (7)
C22—C21—C23	107.49 (18)	C62—C57—C56	108.1 (7)
C12—C21—C23	110.13 (16)	C59—C58—C57	120.0
C22—C21—C24	107.20 (17)	C59—C58—H58	120.0
C12—C21—C24	109.47 (17)	C57—C58—H58	120.0
C23—C21—C24	110.82 (16)	C58—C59—C60	120.0
C21—C22—H22A	109.5	C58—C59—H59	120.0
C21—C22—H22B	109.5	C60—C59—H59	120.0
H22A—C22—H22B	109.5	C57—C62—C63	109.7 (5)
C21—C22—H22C	109.5	C51—C63—C62	103.4 (4)
H22A—C22—H22C	109.5	C51—C63—H63A	111.1
H22B—C22—H22C	109.5	C62—C63—H63A	111.1
C21—C23—H23A	109.5	C51—C63—H63B	111.1
C21—C23—H23B	109.5	C62—C63—H63B	111.1
H23A—C23—H23B	109.5	H63A—C63—H63B	109.0

### Hydrogen-bond geometry (Å, º)

**Table:**

*D*—H···*A*	*D*—H	H···*A*	*D*···*A*	*D*—H···*A*
O4—H4···O3	0.89 (3)	1.76 (3)	2.648 (2)	180 (3)

## References

[bb1] Blessing, R. H. (1995). *Acta Cryst.* A**51**, 33–38. 10.1107/s0108767394005726 7702794

[bb2] Farrugia, L. J. (1999). *J. Appl. Cryst.* **32**, 837–838.

[bb3] Gielens, E. E. C. G., Dijkstra, T. W., Berno, P., Meetsma, A., Hessen, B. & Teuben, J. H. (1999). *J. Organomet. Chem.* **591**, 88–95.

[bb4] Okuda, J., Fokken, S., Kang, H.-C. & Massa, W. (1995). *Chem. Ber.* **128**, 221–227.

[bb5] Sheldrick, G. M. (2008). *Acta Cryst.* A**64**, 112–122. 10.1107/S0108767307043930 18156677

[bb6] Siemens (1995). *SAINT* and *SMART* Siemens Analytical X-ray Instruments Inc., Madison, Wisconsin, USA.

[bb7] Toscano, P. J., James Schermerhorn, E. J., Barren, E., Liu, S. & Zubieta, J. (1998). *J. Coord. Chem.* **43**, 169–185.

